# A new polymorph of 2,6-bis­(trifluoro­meth­yl)benzoic acid

**DOI:** 10.1107/S1600536811011731

**Published:** 2011-04-07

**Authors:** Nick A. Giffin, Arthur D. Hendsbee, Jason D. Masuda

**Affiliations:** a The Maritimes Centre for Green Chemistry and the Department of Chemistry, Saint Mary’s University, 923 Robie Street, Halifax, NS B3H 3C3, Canada

## Abstract

The asymmetric unit of a second polymorph of the title compound, C_9_H_4_F_6_O_2_, contains five independent mol­ecules, which form hydrogen-bonded O—H⋯O dimers about inversion centers. The most significant structural difference between this structure and that of the first polymorph [Tobin & Masuda (2009 ▶). *Acta Cryst.* E**65**, o1217] is the hydrogen-bonded, dimeric orientation of the carb­oxy­lic acid functionalities.

## Related literature

For the first polymorph of the title compound, see: Tobin & Masuda (2009 ▶). For details of the synthesis, see: Dmowski & Piasecka-Macieiewska (1998) ▶. For information on dimeric *versus* catemeric crystal growth in benzoic acids, see: Moorthy *et al.* (2002 ▶). 
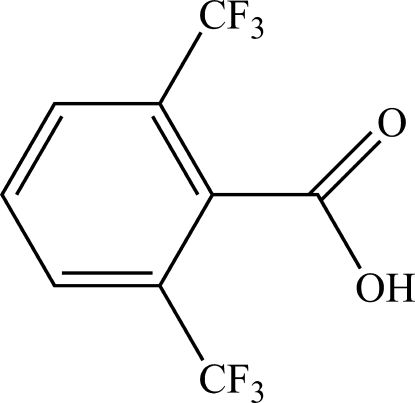


## Experimental

### 

#### Crystal data

C_9_H_4_F_6_O_2_
*M* *_r_* = 258.12
Triclinic, 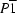

*a* = 10.312 (2) Å
*b* = 11.243 (2) Å
*c* = 21.283 (4) Å
α = 79.565 (3)°
β = 88.961 (3)°
γ = 85.125 (3)°
*V* = 2418.0 (9) Å^3^
*Z* = 10
Mo *K*α radiation
μ = 0.20 mm^−1^
*T* = 100 K
0.14 × 0.11 × 0.11 mm

#### Data collection

Bruker APEXII CCD diffractometer
Absorption correction: multi-scan (*SADABS*; Bruker, 2010 ▶) *T*_min_ = 0.973, *T*_max_ = 0.980
16341 measured reflections
8352 independent reflections
5092 reflections with *I* > 2σ(*I*)
*R*_int_ = 0.046

#### Refinement

*R*[*F* ^2^ > 2σ(*F* ^2^)] = 0.048
*wR*(*F* ^2^) = 0.151
*S* = 0.85
8352 reflections
771 parameters
6 restraints
H-atom parameters constrained
Δρ_max_ = 0.28 e Å^−3^
Δρ_min_ = −0.29 e Å^−3^

### 

Data collection: *APEX2* (Bruker, 2010 ▶); cell refinement: *SAINT* (Bruker, 2010 ▶); data reduction: *SAINT*; program(s) used to solve structure: *SHELXS97* (Sheldrick, 2008 ▶); program(s) used to refine structure: *SHELXL97* (Sheldrick, 2008 ▶); molecular graphics: *ORTEP-3 for Windows* (Farrugia, 1997 ▶) and *Mercury* (Macrae *et al.*, 2006 ▶); software used to prepare material for publication: *SHELXTL* (Sheldrick, 2008 ▶).

## Supplementary Material

Crystal structure: contains datablocks I, global. DOI: 10.1107/S1600536811011731/pv2403sup1.cif

Structure factors: contains datablocks I. DOI: 10.1107/S1600536811011731/pv2403Isup2.hkl

Additional supplementary materials:  crystallographic information; 3D view; checkCIF report

## Figures and Tables

**Table 1:** Hydrogen-bond geometry (Å, °)

*D*—H⋯*A*	*D*—H	H⋯*A*	*D*⋯*A*	*D*—H⋯*A*
O2—H2⋯O1^i^	0.84	1.87	2.701 (4)	172
O4—H4*A*⋯O10^ii^	0.84	1.82	2.657 (3)	174
O6—H6*A*⋯O8^i^	0.84	1.84	2.673 (4)	170
O7—H7⋯O5^i^	0.84	1.80	2.638 (4)	173
O9—H9⋯O3^iii^	0.84	1.81	2.644 (3)	174

Symmetry codes: (i) 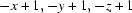; (ii) 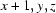; (iii) 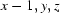.

**Fig. 1.**
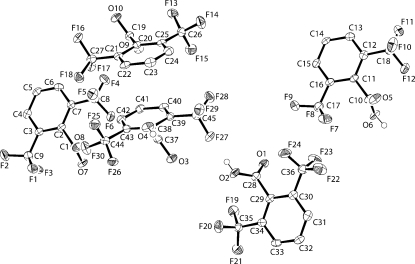
The molecular structure of the title compound, with atom labels and 50% probability displacement ellipsoids for non-H atoms. Aromatic hydrogen atoms are removed for clarity.

**Fig. 2.**
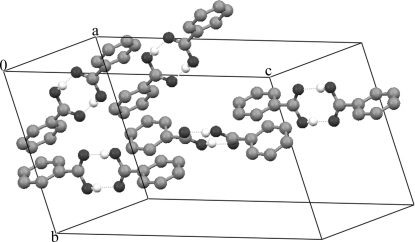
The hydorgen bonding interactions between molecules of the title compound lying about inversion centers. Trifluoromethyl groups and aromatic H atoms are removed for clarity.

## supplementary crystallographic information

### Comment

Recent studies on benzoic acids have indicated a potential for selective crystal polymorph engineering. It is suggested that catemeric or dimeric structures of benzoic acids can be influenced by concentration and the presence of solvent (Moorthy *et al.*, 2002). Crystallization from solvent free oil led to the formation of the hydrogen bound, dimeric form of the title compound.

The molecular structure of the title compound is presented in Fig. 1. The most significant structural difference between this structure and the literature polymorph (Tobin & Masuda, 2009) is the hydrogen bound, dimeric orientation of the carboxylic acid functionalities. The five molecules in the asymmetric unit are defined by O—H···O hydrogen bonds ranging from 2.638 (4)–2.701 (4) Å and angles of 170–174° (Tab. 1 & Fig. 2).

### Experimental

The title compound was prepared following the literature methods (Dmowski & Piasecka-Macieiewska, 1998). The compound was crystallized slowly from the resultant oil forming colorless, block-like crystals.

### Refinement

The H atoms were placed in geometrically idealized positions with C—H and O—H distances = 0.95 and 0.98Å, respectively, with *U*_iso_(H) = 1.2*U*_eq_(C) or 1.5*U*_eq_(O).

Several short contact distances are found for F(2) & F(4) to F(17), F(8) to F(23), F(9) to F(20), F(10) to F(25), F(13) to F(21) and F(14) to F(27). The short contacts are believed to arise from disorder present in the crystal. Modeling this disorder yielded low occupancy which was detrimental to the integrity of the data set upon refinement. In order to obtain satisfactory thermal parameters the use of DELU restraints were applied to fluorine atoms F(4)>F(6) relative to the adjacent C(8) atom.

### Crystal data

**Table:**

C_9_H_4_F_6_O_2_	*Z* = 10
*M**_r_* = 258.12	*F*(000) = 1280
Triclinic, *P*1	*D*_x_ = 1.773 Mg m^−^^3^
Hall symbol: -P 1	Mo *K*α radiation, λ = 0.71073 Å
*a* = 10.312 (2) Å	Cell parameters from 4371 reflections
*b* = 11.243 (2) Å	θ = 2.2–27.4°
*c* = 21.283 (4) Å	µ = 0.20 mm^−^^1^
α = 79.565 (3)°	*T* = 100 K
β = 88.961 (3)°	Block, colourless
γ = 85.125 (3)°	0.14 × 0.11 × 0.11 mm
*V* = 2418.0 (9) Å^3^	

### Data collection

**Table:**

Bruker APEXII CCD diffractometer	8352 independent reflections
Radiation source: fine-focus sealed tube	5092 reflections with *I* > 2σ(*I*)
graphite	*R*_int_ = 0.046
φ and ω scans	θ_max_ = 25.0°, θ_min_ = 1.9°
Absorption correction: multi-scan (*SADABS*; Bruker, 2010)	*h* = −11→12
*T*_min_ = 0.973, *T*_max_ = 0.980	*k* = −13→13
16341 measured reflections	*l* = −25→25

### Refinement

**Table:**

Refinement on *F*^2^	Primary atom site location: structure-invariant direct methods
Least-squares matrix: full	Secondary atom site location: difference Fourier map
*R*[*F*^2^ > 2σ(*F*^2^)] = 0.048	Hydrogen site location: inferred from neighbouring sites
*wR*(*F*^2^) = 0.151	H-atom parameters constrained
*S* = 0.85	*w* = 1/[σ^2^(*F*_o_^2^) + (0.0887*P*)^2^ + 1.2723*P*] where *P* = (*F*_o_^2^ + 2*F*_c_^2^)/3
8352 reflections	(Δ/σ)_max_ = 0.001
771 parameters	Δρ_max_ = 0.28 e Å^−^^3^
6 restraints	Δρ_min_ = −0.29 e Å^−^^3^

### Special details

**Table:**

Geometry. All e.s.d.'s (except the e.s.d. in the dihedral angle between two l.s. planes) are estimated using the full covariance matrix. The cell e.s.d.'s are taken into account individually in the estimation of e.s.d.'s in distances, angles and torsion angles; correlations between e.s.d.'s in cell parameters are only used when they are defined by crystal symmetry. An approximate (isotropic) treatment of cell e.s.d.'s is used for estimating e.s.d.'s involving l.s. planes.
Refinement. Refinement of *F*^2^ against ALL reflections. The weighted *R*-factor *wR* and goodness of fit *S* are based on *F*^2^, conventional *R*-factors *R* are based on *F*, with *F* set to zero for negative *F*^2^. The threshold expression of *F*^2^ > σ(*F*^2^) is used only for calculating *R*-factors(gt) *etc*. and is not relevant to the choice of reflections for refinement. *R*-factors based on *F*^2^ are statistically about twice as large as those based on *F*, and *R*- factors based on ALL data will be even larger.

### Fractional atomic coordinates and isotropic or equivalent isotropic displacement parameters (Å^2^)

**Table:**

	*x*	*y*	*z*	*U*_iso_*/*U*_eq_	
F1	0.2018 (2)	0.7426 (2)	−0.03174 (10)	0.0326 (5)	
C1	0.1804 (4)	0.6502 (3)	0.08995 (16)	0.0210 (8)	
O1	0.5747 (2)	0.5485 (2)	0.55641 (12)	0.0268 (6)	
F2	0.0386 (2)	0.7786 (2)	−0.09609 (10)	0.0391 (6)	
C2	0.0469 (3)	0.6267 (3)	0.06966 (16)	0.0184 (8)	
O2	0.6703 (2)	0.4971 (2)	0.46844 (12)	0.0286 (6)	
H2	0.5936	0.4894	0.4581	0.043*	
F3	0.0525 (2)	0.88431 (19)	−0.02227 (11)	0.0412 (6)	
C3	−0.0026 (3)	0.6834 (3)	0.00950 (17)	0.0207 (8)	
O3	0.8142 (2)	0.2255 (2)	0.30586 (12)	0.0256 (6)	
C4	−0.1242 (4)	0.6592 (3)	−0.00972 (17)	0.0224 (8)	
H4	−0.1558	0.6956	−0.0510	0.027*	
O4	0.7566 (2)	0.0674 (2)	0.26430 (12)	0.0263 (6)	
H4A	0.8359	0.0441	0.2695	0.039*	
C5	−0.1996 (4)	0.5822 (3)	0.03096 (17)	0.0227 (8)	
H5	−0.2831	0.5665	0.0179	0.027*	
O5	0.5766 (2)	0.2035 (2)	0.86826 (13)	0.0313 (6)	
C6	−0.1525 (4)	0.5288 (3)	0.09046 (17)	0.0234 (9)	
H6	−0.2049	0.4770	0.1185	0.028*	
O6	0.4856 (2)	0.3878 (2)	0.87912 (12)	0.0264 (6)	
H6A	0.5603	0.3966	0.8917	0.040*	
C7	−0.0301 (3)	0.5491 (3)	0.11012 (16)	0.0186 (8)	
F7	0.3484 (2)	0.50617 (18)	0.75965 (11)	0.0366 (6)	
O7	0.1849 (2)	0.7599 (2)	0.10138 (12)	0.0250 (6)	
H7	0.2624	0.7721	0.1080	0.037*	
C8	0.0199 (4)	0.4852 (3)	0.17366 (17)	0.0231 (6)	
F8	0.4944 (2)	0.3692 (2)	0.74177 (11)	0.0434 (6)	
O8	0.2712 (2)	0.5717 (2)	0.09473 (11)	0.0241 (6)	
C9	0.0723 (4)	0.7717 (3)	−0.03502 (17)	0.0269 (9)	
F9	0.3243 (3)	0.4099 (2)	0.68321 (11)	0.0577 (8)	
O9	0.0663 (2)	0.1576 (2)	0.31237 (12)	0.0244 (6)	
H9	−0.0132	0.1803	0.3076	0.037*	
F10	0.4166 (2)	0.00770 (19)	0.94653 (11)	0.0433 (7)	
C10	0.4828 (4)	0.2792 (3)	0.86606 (17)	0.0233 (8)	
O10	0.0096 (2)	−0.0017 (2)	0.27140 (12)	0.0266 (6)	
F11	0.4018 (2)	0.17788 (18)	0.98037 (10)	0.0309 (5)	
C11	0.3507 (3)	0.2508 (3)	0.84751 (16)	0.0199 (8)	
F12	0.2413 (2)	0.0653 (2)	0.99315 (10)	0.0424 (6)	
C12	0.2800 (4)	0.1666 (3)	0.88805 (16)	0.0218 (8)	
C13	0.1607 (4)	0.1366 (3)	0.87036 (17)	0.0252 (9)	
H13	0.1138	0.0803	0.8987	0.030*	
F13	0.0824 (2)	−0.0491 (2)	0.41419 (10)	0.0353 (6)	
C14	0.1091 (4)	0.1894 (3)	0.81057 (17)	0.0224 (8)	
H14	0.0270	0.1691	0.7980	0.027*	
F14	0.2542 (2)	−0.1135 (2)	0.46976 (10)	0.0400 (6)	
C15	0.1785 (4)	0.2714 (3)	0.76988 (17)	0.0234 (8)	
H15	0.1441	0.3064	0.7290	0.028*	
F15	0.2106 (2)	0.07786 (19)	0.43794 (10)	0.0370 (6)	
C16	0.2977 (4)	0.3034 (3)	0.78793 (16)	0.0217 (8)	
F16	0.1837 (2)	−0.03585 (18)	0.15962 (9)	0.0278 (5)	
C17	0.3656 (4)	0.3974 (4)	0.74290 (18)	0.0299 (9)	
F17	0.1763 (2)	0.14663 (17)	0.17769 (9)	0.0273 (5)	
C18	0.3351 (4)	0.1034 (3)	0.95232 (18)	0.0278 (9)	
F18	0.3479 (2)	0.07010 (19)	0.13671 (9)	0.0310 (5)	
C19	0.0905 (4)	0.0543 (3)	0.29297 (16)	0.0211 (8)	
F19	0.6650 (2)	0.7594 (2)	0.45210 (10)	0.0356 (6)	
C20	0.2308 (3)	0.0048 (3)	0.30006 (16)	0.0192 (8)	
F20	0.8213 (2)	0.6977 (2)	0.39510 (9)	0.0365 (6)	
C21	0.3090 (3)	−0.0021 (3)	0.24565 (16)	0.0195 (8)	
F21	0.8352 (2)	0.85782 (18)	0.43459 (10)	0.0361 (6)	
C22	0.4375 (4)	−0.0487 (3)	0.25249 (18)	0.0223 (8)	
H22	0.4903	−0.0511	0.2156	0.027*	
F22	0.9052 (2)	0.3168 (2)	0.66535 (12)	0.0454 (7)	
C23	0.4900 (4)	−0.0917 (3)	0.31231 (18)	0.0255 (9)	
H23	0.5779	−0.1248	0.3164	0.031*	
F23	0.7250 (2)	0.4195 (2)	0.67639 (11)	0.0417 (6)	
C24	0.4143 (4)	−0.0866 (3)	0.36632 (18)	0.0261 (9)	
H24	0.4503	−0.1167	0.4075	0.031*	
F24	0.7544 (2)	0.32825 (19)	0.59631 (11)	0.0419 (6)	
C25	0.2858 (3)	−0.0376 (3)	0.36039 (16)	0.0202 (8)	
F25	0.6092 (2)	0.14615 (19)	0.14326 (10)	0.0337 (5)	
C26	0.2081 (4)	−0.0306 (3)	0.42061 (18)	0.0273 (9)	
F26	0.7286 (2)	0.28146 (19)	0.16435 (10)	0.0320 (5)	
C27	0.2537 (4)	0.0439 (3)	0.18000 (17)	0.0228 (8)	
F27	0.6488 (2)	0.26378 (18)	0.41950 (9)	0.0277 (5)	
C28	0.6694 (4)	0.5377 (3)	0.52244 (17)	0.0237 (8)	
F28	0.4893 (2)	0.1538 (2)	0.44626 (9)	0.0325 (5)	
C29	0.8011 (3)	0.5702 (3)	0.54062 (16)	0.0198 (8)	
F29	0.6595 (2)	0.07953 (17)	0.40289 (9)	0.0281 (5)	
C30	0.8664 (4)	0.5031 (3)	0.59459 (17)	0.0226 (8)	
F30	0.5517 (2)	0.3352 (2)	0.11093 (10)	0.0409 (6)	
C31	0.9828 (4)	0.5382 (3)	0.61349 (17)	0.0259 (9)	
H31	1.0254	0.4930	0.6505	0.031*	
C32	1.0379 (4)	0.6378 (3)	0.57941 (18)	0.0255 (9)	
H32	1.1180	0.6610	0.5927	0.031*	
C33	0.9757 (4)	0.7032 (3)	0.52601 (17)	0.0238 (9)	
H33	1.0136	0.7716	0.5023	0.029*	
C34	0.8579 (4)	0.6706 (3)	0.50624 (16)	0.0210 (8)	
C35	0.7957 (4)	0.7459 (3)	0.44775 (17)	0.0245 (9)	
C36	0.8120 (4)	0.3929 (3)	0.63253 (18)	0.0287 (9)	
C37	0.7321 (4)	0.1698 (3)	0.28488 (16)	0.0205 (8)	
C38	0.5913 (3)	0.2176 (3)	0.28029 (16)	0.0189 (8)	
C39	0.5192 (4)	0.2240 (3)	0.33629 (16)	0.0197 (8)	
C40	0.3908 (4)	0.2727 (3)	0.33246 (17)	0.0226 (8)	
H40	0.3421	0.2763	0.3705	0.027*	
C41	0.3330 (4)	0.3163 (3)	0.27363 (17)	0.0238 (8)	
H41	0.2454	0.3504	0.2715	0.029*	
C42	0.4026 (4)	0.3101 (3)	0.21823 (17)	0.0244 (9)	
H42	0.3626	0.3394	0.1779	0.029*	
C43	0.5319 (4)	0.2609 (3)	0.22122 (16)	0.0216 (8)	
C44	0.6044 (4)	0.2552 (3)	0.16000 (17)	0.0254 (9)	
C45	0.5793 (4)	0.1803 (3)	0.40084 (17)	0.0237 (8)	
F4	0.1004 (2)	0.38607 (18)	0.16895 (10)	0.0319 (5)	
F5	−0.0752 (2)	0.4452 (2)	0.21359 (10)	0.0356 (5)	
F6	0.0871 (2)	0.55444 (18)	0.20437 (9)	0.0289 (5)	

### Atomic displacement parameters (Å^2^)

**Table:**

	*U*^11^	*U*^22^	*U*^33^	*U*^12^	*U*^13^	*U*^23^
F1	0.0198 (13)	0.0469 (14)	0.0305 (12)	−0.0067 (10)	0.0055 (10)	−0.0040 (10)
C1	0.020 (2)	0.0212 (18)	0.0229 (19)	−0.0016 (16)	0.0007 (16)	−0.0061 (15)
O1	0.0138 (14)	0.0371 (15)	0.0322 (14)	−0.0036 (11)	0.0023 (12)	−0.0127 (12)
F2	0.0322 (14)	0.0588 (15)	0.0219 (11)	−0.0052 (12)	−0.0015 (10)	0.0054 (11)
C2	0.0151 (19)	0.0191 (17)	0.0228 (18)	−0.0004 (14)	0.0000 (15)	−0.0084 (15)
O2	0.0157 (14)	0.0436 (16)	0.0324 (15)	−0.0070 (13)	0.0011 (12)	−0.0205 (13)
F3	0.0440 (15)	0.0242 (11)	0.0533 (15)	−0.0063 (10)	0.0131 (12)	−0.0006 (11)
C3	0.014 (2)	0.0228 (18)	0.0271 (19)	−0.0001 (15)	0.0019 (16)	−0.0098 (16)
O3	0.0168 (14)	0.0280 (13)	0.0340 (15)	−0.0026 (11)	−0.0016 (12)	−0.0103 (12)
C4	0.021 (2)	0.0225 (18)	0.0248 (19)	0.0048 (16)	−0.0030 (16)	−0.0092 (16)
O4	0.0139 (14)	0.0247 (13)	0.0415 (15)	0.0025 (11)	−0.0002 (13)	−0.0113 (12)
C5	0.016 (2)	0.0237 (18)	0.031 (2)	−0.0006 (15)	−0.0031 (17)	−0.0123 (17)
O5	0.0164 (14)	0.0317 (14)	0.0505 (17)	0.0031 (12)	−0.0024 (13)	−0.0217 (13)
C6	0.019 (2)	0.0204 (18)	0.031 (2)	−0.0014 (15)	0.0047 (17)	−0.0053 (16)
O6	0.0175 (15)	0.0262 (13)	0.0395 (15)	−0.0010 (11)	−0.0075 (13)	−0.0158 (12)
C7	0.014 (2)	0.0190 (17)	0.0235 (18)	0.0012 (15)	0.0011 (15)	−0.0081 (15)
F7	0.0372 (14)	0.0255 (11)	0.0470 (14)	−0.0088 (10)	0.0025 (12)	−0.0033 (11)
O7	0.0173 (14)	0.0251 (13)	0.0356 (15)	−0.0029 (11)	−0.0036 (12)	−0.0128 (12)
C8	0.0206 (19)	0.0219 (16)	0.0268 (19)	−0.0002 (10)	−0.0001 (13)	−0.0054 (14)
F8	0.0318 (15)	0.0483 (14)	0.0512 (15)	−0.0117 (11)	0.0169 (12)	−0.0099 (12)
O8	0.0149 (14)	0.0250 (13)	0.0333 (14)	0.0007 (11)	−0.0050 (11)	−0.0085 (11)
C9	0.023 (2)	0.031 (2)	0.027 (2)	−0.0001 (17)	0.0004 (17)	−0.0053 (17)
F9	0.080 (2)	0.0733 (18)	0.0228 (13)	−0.0463 (16)	−0.0016 (13)	0.0012 (12)
O9	0.0163 (14)	0.0250 (13)	0.0331 (14)	0.0021 (11)	0.0002 (12)	−0.0104 (11)
F10	0.0571 (17)	0.0273 (12)	0.0453 (14)	0.0187 (12)	−0.0244 (13)	−0.0143 (11)
C10	0.018 (2)	0.0258 (19)	0.030 (2)	−0.0022 (16)	−0.0014 (17)	−0.0157 (17)
O10	0.0163 (14)	0.0281 (13)	0.0383 (15)	−0.0014 (11)	−0.0046 (12)	−0.0135 (12)
F11	0.0327 (14)	0.0293 (11)	0.0320 (12)	0.0006 (10)	−0.0128 (10)	−0.0097 (10)
C11	0.017 (2)	0.0196 (17)	0.0255 (19)	−0.0017 (15)	0.0004 (16)	−0.0102 (15)
F12	0.0482 (16)	0.0463 (14)	0.0290 (12)	−0.0138 (12)	−0.0048 (12)	0.0080 (11)
C12	0.023 (2)	0.0177 (17)	0.0257 (19)	0.0045 (15)	−0.0020 (17)	−0.0081 (15)
C13	0.025 (2)	0.0226 (19)	0.029 (2)	−0.0043 (16)	0.0023 (18)	−0.0062 (16)
F13	0.0236 (13)	0.0486 (14)	0.0326 (12)	−0.0120 (11)	0.0047 (10)	−0.0004 (11)
C14	0.017 (2)	0.0238 (18)	0.029 (2)	−0.0055 (15)	−0.0042 (16)	−0.0088 (16)
F14	0.0439 (15)	0.0475 (14)	0.0246 (12)	−0.0004 (12)	−0.0034 (11)	0.0023 (11)
C15	0.025 (2)	0.0231 (18)	0.0233 (19)	−0.0012 (16)	−0.0055 (17)	−0.0084 (16)
F15	0.0412 (15)	0.0362 (13)	0.0377 (13)	−0.0073 (11)	0.0100 (11)	−0.0167 (11)
C16	0.019 (2)	0.0232 (18)	0.0255 (19)	−0.0037 (15)	0.0017 (16)	−0.0099 (16)
F16	0.0269 (13)	0.0299 (11)	0.0304 (11)	−0.0039 (10)	−0.0054 (10)	−0.0142 (10)
C17	0.029 (2)	0.033 (2)	0.030 (2)	−0.0056 (18)	−0.0006 (18)	−0.0095 (18)
F17	0.0308 (13)	0.0216 (11)	0.0284 (11)	0.0073 (9)	−0.0059 (10)	−0.0055 (9)
C18	0.028 (2)	0.0235 (19)	0.033 (2)	0.0009 (17)	−0.0071 (19)	−0.0080 (17)
F18	0.0270 (13)	0.0392 (12)	0.0258 (11)	−0.0035 (10)	0.0032 (10)	−0.0035 (10)
C19	0.019 (2)	0.0226 (18)	0.0232 (19)	−0.0017 (16)	0.0003 (16)	−0.0074 (16)
F19	0.0186 (12)	0.0465 (14)	0.0371 (13)	0.0052 (10)	−0.0028 (10)	0.0008 (11)
C20	0.019 (2)	0.0149 (16)	0.0247 (19)	−0.0022 (14)	−0.0017 (16)	−0.0056 (15)
F20	0.0402 (15)	0.0470 (14)	0.0243 (12)	−0.0007 (11)	0.0013 (10)	−0.0129 (11)
C21	0.018 (2)	0.0140 (16)	0.0257 (19)	0.0011 (15)	−0.0031 (16)	−0.0038 (15)
F21	0.0414 (15)	0.0264 (11)	0.0381 (13)	−0.0028 (10)	−0.0032 (11)	0.0008 (10)
C22	0.019 (2)	0.0167 (17)	0.034 (2)	−0.0030 (15)	0.0003 (17)	−0.0099 (16)
F22	0.0348 (15)	0.0353 (13)	0.0569 (16)	0.0032 (11)	−0.0033 (13)	0.0139 (12)
C23	0.016 (2)	0.0206 (18)	0.039 (2)	0.0039 (15)	−0.0028 (18)	−0.0049 (17)
F23	0.0372 (15)	0.0417 (13)	0.0444 (14)	−0.0084 (11)	0.0167 (12)	−0.0025 (11)
C24	0.028 (2)	0.0179 (18)	0.032 (2)	−0.0003 (16)	−0.0050 (18)	−0.0028 (16)
F24	0.0456 (16)	0.0279 (12)	0.0536 (15)	−0.0161 (11)	−0.0043 (13)	−0.0044 (11)
C25	0.020 (2)	0.0166 (17)	0.0254 (19)	−0.0055 (15)	0.0000 (16)	−0.0054 (15)
F25	0.0370 (14)	0.0351 (12)	0.0335 (12)	−0.0105 (10)	0.0078 (11)	−0.0152 (10)
C26	0.026 (2)	0.029 (2)	0.027 (2)	−0.0062 (17)	−0.0065 (18)	0.0004 (17)
F26	0.0302 (14)	0.0381 (12)	0.0314 (12)	−0.0174 (10)	0.0097 (10)	−0.0101 (10)
C27	0.021 (2)	0.0201 (18)	0.029 (2)	−0.0013 (16)	0.0031 (17)	−0.0084 (16)
F27	0.0247 (12)	0.0306 (11)	0.0311 (11)	−0.0046 (10)	−0.0071 (10)	−0.0123 (10)
C28	0.019 (2)	0.0261 (19)	0.027 (2)	−0.0002 (16)	0.0002 (17)	−0.0076 (17)
F28	0.0272 (13)	0.0429 (13)	0.0253 (11)	−0.0023 (10)	0.0032 (10)	−0.0008 (10)
C29	0.0131 (19)	0.0220 (18)	0.0270 (19)	0.0010 (15)	0.0011 (16)	−0.0126 (16)
F29	0.0290 (13)	0.0250 (11)	0.0289 (11)	0.0071 (10)	−0.0052 (10)	−0.0048 (9)
C30	0.019 (2)	0.0233 (18)	0.027 (2)	−0.0003 (16)	0.0009 (17)	−0.0093 (16)
F30	0.0473 (16)	0.0472 (14)	0.0243 (12)	0.0005 (12)	0.0010 (11)	0.0015 (11)
C31	0.023 (2)	0.0263 (19)	0.029 (2)	0.0050 (17)	−0.0053 (17)	−0.0077 (17)
C32	0.018 (2)	0.0262 (19)	0.035 (2)	−0.0029 (16)	−0.0033 (17)	−0.0102 (17)
C33	0.019 (2)	0.0214 (18)	0.033 (2)	−0.0039 (16)	0.0060 (17)	−0.0087 (17)
C34	0.021 (2)	0.0201 (18)	0.0233 (19)	−0.0019 (15)	0.0034 (16)	−0.0076 (15)
C35	0.021 (2)	0.029 (2)	0.025 (2)	−0.0047 (16)	0.0023 (17)	−0.0075 (17)
C36	0.023 (2)	0.028 (2)	0.033 (2)	−0.0025 (18)	−0.0043 (19)	−0.0012 (18)
C37	0.019 (2)	0.0220 (18)	0.0214 (18)	−0.0027 (16)	0.0034 (16)	−0.0051 (16)
C38	0.0130 (19)	0.0169 (17)	0.0275 (19)	−0.0016 (14)	−0.0010 (16)	−0.0059 (15)
C39	0.022 (2)	0.0152 (17)	0.0226 (18)	−0.0023 (15)	−0.0023 (16)	−0.0045 (15)
C40	0.019 (2)	0.0215 (18)	0.029 (2)	−0.0015 (15)	0.0029 (17)	−0.0084 (16)
C41	0.017 (2)	0.0233 (19)	0.031 (2)	−0.0004 (16)	−0.0049 (17)	−0.0068 (16)
C42	0.026 (2)	0.0194 (18)	0.027 (2)	−0.0025 (16)	−0.0070 (17)	−0.0023 (16)
C43	0.022 (2)	0.0209 (18)	0.0232 (19)	−0.0049 (16)	0.0019 (16)	−0.0050 (15)
C44	0.026 (2)	0.025 (2)	0.026 (2)	−0.0067 (17)	0.0010 (17)	−0.0023 (17)
C45	0.018 (2)	0.0278 (19)	0.027 (2)	0.0003 (16)	0.0031 (17)	−0.0093 (17)
F4	0.0323 (13)	0.0240 (10)	0.0372 (12)	0.0096 (9)	−0.0095 (10)	−0.0040 (9)
F5	0.0306 (12)	0.0427 (13)	0.0286 (12)	−0.0050 (9)	0.0018 (9)	0.0067 (10)
F6	0.0317 (13)	0.0310 (11)	0.0250 (11)	−0.0016 (9)	−0.0067 (9)	−0.0076 (9)

### Geometric parameters (Å, °)

**Table:**

F1—C9	1.347 (4)	C16—C17	1.503 (5)
C1—O8	1.223 (4)	F16—C27	1.332 (4)
C1—O7	1.305 (4)	F17—C27	1.340 (4)
C1—C2	1.509 (5)	F18—C27	1.341 (4)
O1—C28	1.215 (4)	C19—C20	1.505 (5)
F2—C9	1.339 (4)	F19—C35	1.347 (4)
C2—C7	1.398 (5)	C20—C25	1.399 (5)
C2—C3	1.407 (5)	C20—C21	1.409 (5)
O2—C28	1.310 (4)	F20—C35	1.343 (4)
O2—H2	0.8400	C21—C22	1.384 (5)
F3—C9	1.341 (4)	C21—C27	1.504 (5)
C3—C4	1.389 (5)	F21—C35	1.336 (4)
C3—C9	1.498 (5)	C22—C23	1.380 (5)
O3—C37	1.231 (4)	C22—H22	0.9500
C4—C5	1.386 (5)	F22—C36	1.346 (4)
C4—H4	0.9500	C23—C24	1.384 (5)
O4—C37	1.308 (4)	C23—H23	0.9500
O4—H4A	0.8400	F23—C36	1.338 (4)
C5—C6	1.377 (5)	C24—C25	1.390 (5)
C5—H5	0.9500	C24—H24	0.9500
O5—C10	1.229 (4)	F24—C36	1.330 (5)
C6—C7	1.387 (5)	C25—C26	1.510 (5)
C6—H6	0.9500	F25—C44	1.335 (4)
O6—C10	1.303 (4)	F26—C44	1.347 (4)
O6—H6A	0.8400	F27—C45	1.345 (4)
C7—C8	1.490 (5)	C28—C29	1.511 (5)
F7—C17	1.332 (4)	F28—C45	1.340 (4)
O7—H7	0.8400	C29—C34	1.396 (5)
C8—F5	1.338 (4)	C29—C30	1.404 (5)
C8—F6	1.345 (4)	F29—C45	1.340 (4)
C8—F4	1.350 (4)	C30—C31	1.383 (5)
F8—C17	1.340 (5)	C30—C36	1.497 (5)
F9—C17	1.327 (4)	F30—C44	1.339 (4)
O9—C19	1.305 (4)	C31—C32	1.379 (5)
O9—H9	0.8400	C31—H31	0.9500
F10—C18	1.330 (4)	C32—C33	1.373 (5)
C10—C11	1.500 (5)	C32—H32	0.9500
O10—C19	1.229 (4)	C33—C34	1.392 (5)
F11—C18	1.351 (4)	C33—H33	0.9500
C11—C16	1.398 (5)	C34—C35	1.494 (5)
C11—C12	1.404 (5)	C37—C38	1.503 (5)
F12—C18	1.334 (4)	C38—C43	1.396 (5)
C12—C13	1.379 (5)	C38—C39	1.403 (5)
C12—C18	1.519 (5)	C39—C40	1.387 (5)
C13—C14	1.397 (5)	C39—C45	1.499 (5)
C13—H13	0.9500	C40—C41	1.385 (5)
F13—C26	1.344 (4)	C40—H40	0.9500
C14—C15	1.383 (5)	C41—C42	1.379 (5)
C14—H14	0.9500	C41—H41	0.9500
F14—C26	1.334 (4)	C42—C43	1.397 (5)
C15—C16	1.390 (5)	C42—H42	0.9500
C15—H15	0.9500	C43—C44	1.499 (5)
F15—C26	1.338 (4)		
			
O8—C1—O7	126.0 (3)	C24—C23—H23	120.0
O8—C1—C2	121.5 (3)	C23—C24—C25	120.1 (4)
O7—C1—C2	112.5 (3)	C23—C24—H24	120.0
C7—C2—C3	118.8 (3)	C25—C24—H24	120.0
C7—C2—C1	120.9 (3)	C24—C25—C20	120.6 (3)
C3—C2—C1	120.3 (3)	C24—C25—C26	118.3 (3)
C28—O2—H2	109.5	C20—C25—C26	121.1 (3)
C4—C3—C2	120.2 (3)	F14—C26—F15	106.9 (3)
C4—C3—C9	118.3 (3)	F14—C26—F13	106.6 (3)
C2—C3—C9	121.5 (3)	F15—C26—F13	107.0 (3)
C5—C4—C3	120.4 (3)	F14—C26—C25	111.8 (3)
C5—C4—H4	119.8	F15—C26—C25	111.8 (3)
C3—C4—H4	119.8	F13—C26—C25	112.4 (3)
C37—O4—H4A	109.5	F16—C27—F17	107.1 (3)
C6—C5—C4	119.5 (4)	F16—C27—F18	107.1 (3)
C6—C5—H5	120.2	F17—C27—F18	106.1 (3)
C4—C5—H5	120.2	F16—C27—C21	112.7 (3)
C5—C6—C7	121.2 (3)	F17—C27—C21	111.9 (3)
C5—C6—H6	119.4	F18—C27—C21	111.5 (3)
C7—C6—H6	119.4	O1—C28—O2	125.4 (4)
C10—O6—H6A	109.5	O1—C28—C29	121.2 (3)
C6—C7—C2	119.9 (3)	O2—C28—C29	113.4 (3)
C6—C7—C8	119.6 (3)	C34—C29—C30	118.4 (3)
C2—C7—C8	120.4 (3)	C34—C29—C28	121.1 (3)
C1—O7—H7	109.5	C30—C29—C28	120.4 (3)
F5—C8—F6	106.1 (3)	C31—C30—C29	120.1 (3)
F5—C8—F4	105.6 (3)	C31—C30—C36	118.9 (3)
F6—C8—F4	106.2 (3)	C29—C30—C36	120.9 (3)
F5—C8—C7	112.7 (3)	C32—C31—C30	121.0 (4)
F6—C8—C7	113.6 (3)	C32—C31—H31	119.5
F4—C8—C7	112.0 (3)	C30—C31—H31	119.5
F2—C9—F3	106.6 (3)	C33—C32—C31	119.3 (4)
F2—C9—F1	106.6 (3)	C33—C32—H32	120.3
F3—C9—F1	106.5 (3)	C31—C32—H32	120.3
F2—C9—C3	112.1 (3)	C32—C33—C34	120.8 (3)
F3—C9—C3	111.8 (3)	C32—C33—H33	119.6
F1—C9—C3	112.7 (3)	C34—C33—H33	119.6
C19—O9—H9	109.5	C33—C34—C29	120.2 (3)
O5—C10—O6	125.4 (3)	C33—C34—C35	118.0 (3)
O5—C10—C11	120.7 (3)	C29—C34—C35	121.8 (3)
O6—C10—C11	113.9 (3)	F21—C35—F20	105.8 (3)
C16—C11—C12	118.3 (3)	F21—C35—F19	106.2 (3)
C16—C11—C10	120.6 (3)	F20—C35—F19	105.7 (3)
C12—C11—C10	121.0 (3)	F21—C35—C34	113.0 (3)
C13—C12—C11	121.4 (3)	F20—C35—C34	112.7 (3)
C13—C12—C18	118.1 (3)	F19—C35—C34	112.9 (3)
C11—C12—C18	120.5 (4)	F24—C36—F23	107.2 (3)
C12—C13—C14	119.8 (3)	F24—C36—F22	106.1 (3)
C12—C13—H13	120.1	F23—C36—F22	105.4 (3)
C14—C13—H13	120.1	F24—C36—C30	112.9 (3)
C15—C14—C13	119.4 (4)	F23—C36—C30	112.9 (3)
C15—C14—H14	120.3	F22—C36—C30	111.8 (3)
C13—C14—H14	120.3	O3—C37—O4	124.8 (3)
C14—C15—C16	121.0 (3)	O3—C37—C38	121.1 (3)
C14—C15—H15	119.5	O4—C37—C38	114.0 (3)
C16—C15—H15	119.5	C43—C38—C39	119.0 (3)
C15—C16—C11	120.1 (3)	C43—C38—C37	121.3 (3)
C15—C16—C17	118.6 (3)	C39—C38—C37	119.7 (3)
C11—C16—C17	121.3 (3)	C40—C39—C38	120.0 (3)
F9—C17—F7	107.2 (3)	C40—C39—C45	119.0 (3)
F9—C17—F8	106.7 (3)	C38—C39—C45	121.0 (3)
F7—C17—F8	106.5 (3)	C41—C40—C39	120.5 (3)
F9—C17—C16	112.5 (3)	C41—C40—H40	119.7
F7—C17—C16	111.9 (3)	C39—C40—H40	119.7
F8—C17—C16	111.7 (3)	C42—C41—C40	120.0 (3)
F10—C18—F12	107.9 (3)	C42—C41—H41	120.0
F10—C18—F11	107.1 (3)	C40—C41—H41	120.0
F12—C18—F11	106.6 (3)	C41—C42—C43	120.2 (3)
F10—C18—C12	111.5 (3)	C41—C42—H42	119.9
F12—C18—C12	111.7 (3)	C43—C42—H42	119.9
F11—C18—C12	111.9 (3)	C38—C43—C42	120.2 (3)
O10—C19—O9	125.4 (3)	C38—C43—C44	121.0 (3)
O10—C19—C20	120.9 (3)	C42—C43—C44	118.7 (3)
O9—C19—C20	113.7 (3)	F25—C44—F30	106.9 (3)
C25—C20—C21	118.5 (3)	F25—C44—F26	106.5 (3)
C25—C20—C19	121.1 (3)	F30—C44—F26	106.4 (3)
C21—C20—C19	120.4 (3)	F25—C44—C43	113.2 (3)
C22—C21—C20	120.1 (3)	F30—C44—C43	112.1 (3)
C22—C21—C27	119.8 (3)	F26—C44—C43	111.2 (3)
C20—C21—C27	120.0 (3)	F29—C45—F28	106.4 (3)
C23—C22—C21	120.7 (3)	F29—C45—F27	106.9 (3)
C23—C22—H22	119.6	F28—C45—F27	106.2 (3)
C21—C22—H22	119.6	F29—C45—C39	112.6 (3)
C22—C23—C24	120.0 (3)	F28—C45—C39	111.9 (3)
C22—C23—H23	120.0	F27—C45—C39	112.4 (3)

### Hydrogen-bond geometry (Å, °)

**Table:**

*D*—H···*A*	*D*—H	H···*A*	*D*···*A*	*D*—H···*A*
O2—H2···O1^i^	0.84	1.87	2.701 (4)	172
O4—H4A···O10^ii^	0.84	1.82	2.657 (3)	174
O6—H6A···O8^i^	0.84	1.84	2.673 (4)	170
O7—H7···O5^i^	0.84	1.80	2.638 (4)	173
O9—H9···O3^iii^	0.84	1.81	2.644 (3)	174

Symmetry codes: (i) −*x*+1, −*y*+1, −*z*+1; (ii) *x*+1, *y*, *z*; (iii) *x*−1, *y*, *z*.
